# Platform‐Specific Learning Curves in Robotic‐Assisted Total Knee Arthroplasty: A Systematic Review

**DOI:** 10.1111/os.70304

**Published:** 2026-04-16

**Authors:** Ryhan Divyang Patel, Praneshraja Ganesaraja, Kapil Sugand, Sree Kanakala, Indi Gupte, Srikar Reddy Namireddy, Saran Singh Gill

**Affiliations:** ^1^ Imperial College London London UK

**Keywords:** learning curve, operative time, robotic platforms, robotic‐assisted knee arthroplasty, surgical proficiency, total knee replacement

## Abstract

Robotic‐assisted total knee arthroplasty (raTKA) has seen widespread adoption due to its potential to enhance surgical precision and implant alignment. However, learning curves (LCs) for different robotic platforms remain poorly characterized, complicating training and safe implementation. This systematic review quantified the LC across major raTKA systems, focusing on operative time and the number of cases required to achieve procedural proficiency. A systematic search was conducted on December 16, 2024, across MEDLINE, Embase, Scopus, Web of Science, and CENTRAL, following PRISMA 2020 guidelines (PROSPERO: CRD420251026692). Studies reporting original data on LCs in raTKAs were included. Outcomes were operative time, radiographic alignment, complication rates, and patient‐reported outcome measures (PROMs). Due to methodological heterogeneity, meta‐analysis was not performed; weighted means were calculated where appropriate. Forty studies were included, comprising 10,533 procedures across nine robotic platforms. Operative time, reported in 38 studies, was the primary LC metric. Cases required to reach proficiency ranged from 2 to 73. Stratified analysis showed proficiency after a mean of 18.4 cases for NAVIO (mean time 81.9 min), 29.5 cases for ROSA (85.5 min), and 34.2 cases for MAKO (82.0 min). Radiographic accuracy and complication rates remained stable throughout. PROMs were underreported and inconsistent, limiting conclusions. The LC for raTKA is platform dependent. Inconsistent reporting of radiographic and safety outcomes, especially with ROSA, limited secondary endpoint analysis. These findings highlight the need for standardized LC definitions and robust comparative studies to guide training, accreditation, and safe clinical integration.

## Graphical abstract

This systematic review compared learning curves for robotic‐assisted total knee arthroplasty across MAKO, NAVIO, and ROSA platforms. NAVIO required the fewest cases to proficiency, while radiographic accuracy and complication rates remained stable. Patient‐reported outcomes were inconsistently reported.

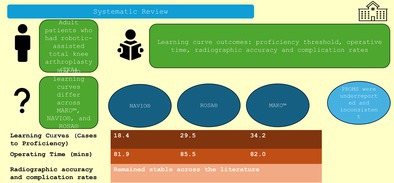

## 1 Introduction

Osteoarthritis (OA) represents a significant global health burden, affecting approximately 600 million people worldwide, with the prevalence of knee OA alone projected to increase by 74.9% by 2050 [1, 2, 3, 4, 5]. Knee OA is initially managed conservatively, often through a combination of physiotherapy and analgesia. However, if conservative management fails surgical intervention is indicated for advanced or end stage OA [6, 7].

Total knee arthroplasty (TKA) is the traditional surgical treatment for severe knee OA, offering significant improvements in pain relief, joint function, and quality of life [8]. Despite its effectiveness, up to 10% of patients report dissatisfaction following manual TKA (mTKA), often due to suboptimal outcomes or refractory pain [9, 10]. In response, robotic‐assisted TKA (raTKA) has been introduced as a potential solution, aiming to improve surgical precision and consistency [11, 12, 13, 14, 15, 16, 17, 18].

Ra‐TKA systems can be classified by the degree of surgeon involvement: autonomous, semi‐active, or passive [19]. In autonomous systems, the robot performs tasks independently, while passive systems act purely as surgical tools with no independent function. Semi‐automatic systems, the most prevalent, offer intraoperative feedback and constraints but rely on the surgeon for execution and decision‐making. However, their adoption presents a learning curve (LC), the complexity of which can affect training, outcomes, and resource allocation [20]. Quantifying this LC is particularly important considering increasing NHS investment in robotic technologies, and the shift toward competency‐based surgical training frameworks [21].

Despite growing interest in raTKA, the literature evaluating subsequent LCs remains limited. Existing studies often focus on individual robotic platforms, involve small cohorts, use varying definitions of proficiency, or assess a narrow range of outcome measures [17, 20, 22, 23, 24, 25, 26]. Therefore, this systematic review aims to synthesize existing evidence using a range of key metrics such as operative time, radiographic and surgical accuracy, complication rates, and patient‐reported outcome measures (PROMs).

## 2 Methods

### 2.1 Search Methodology

**FIGURE 1:**
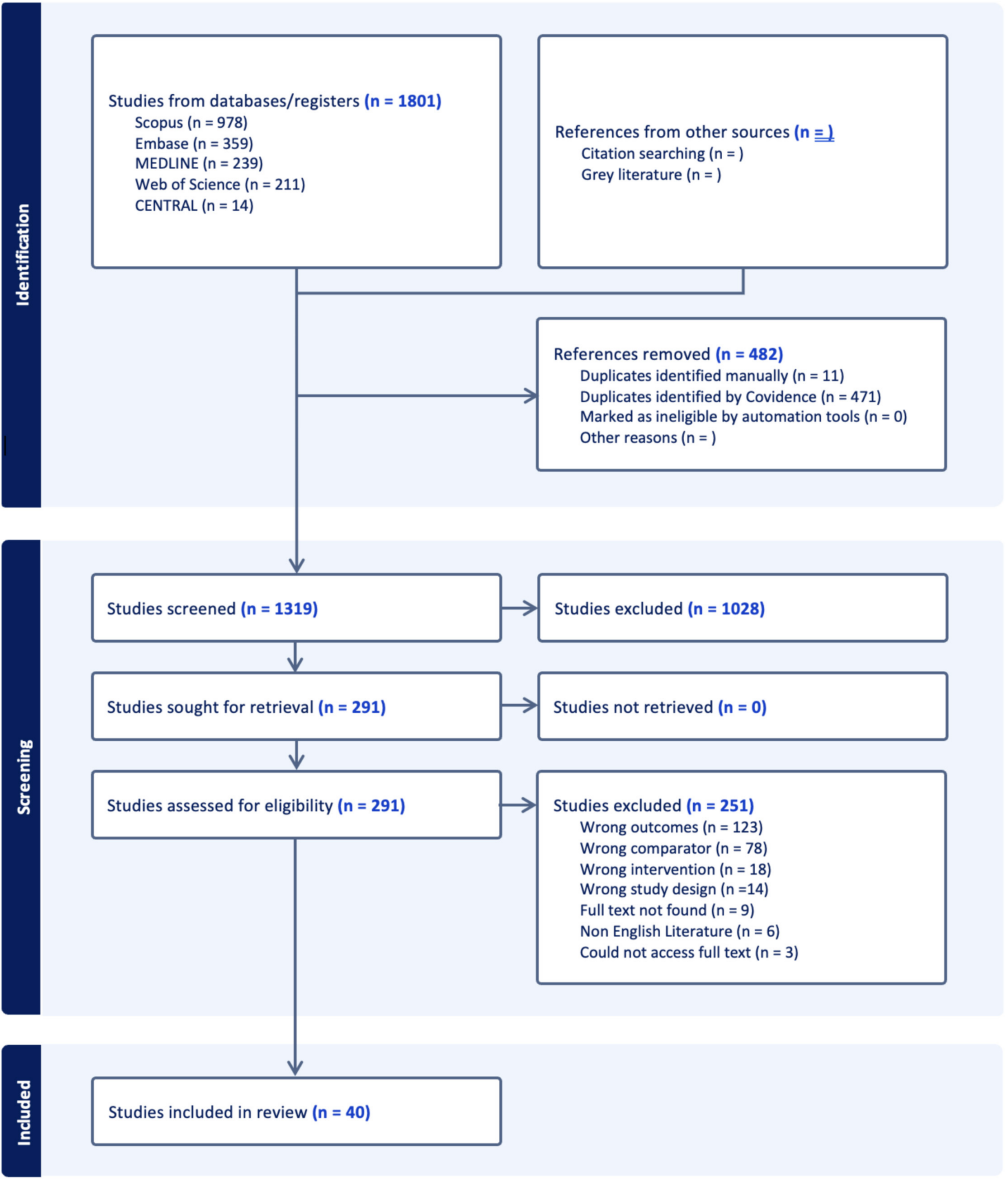
PRISMA Flow Chart showing screening pathway.

This systematic review was conducted following the Cochrane Collaboration guidelines and reported in accordance with the PRISMA 2020 framework [27]. The review protocol was prospectively registered with PROSPERO (CRD420251026692). A comprehensive literature search was performed on December 16th, 2024, across five databases: MEDLINE, Embase, Scopus, Web of Science, and CENTRAL. The search strategy was developed to investigate whether LCs in robotic‐assisted total knee arthroplasty (raTKA) vary according to the robotic platform used. Full database‐specific search strings are available in Table S1. The study selection process is illustrated in the PRISMA flow diagram (Figure 1).

### 2.2 Screening and Eligibility Criteria

All identified records were imported into COVIDENCE for de‐duplication and screening. Title and abstract screening were performed independently by two reviewers (RDP, PG). Full‐text screening of potentially eligible articles was conducted independently by two reviewers. Discrepancies were resolved by consensus, with arbitration by the senior author (SSG) as needed. Studies were included if they were original primary research articles published in English that investigated LCs in robotic‐assisted total knee arthroplasty (raTKA) using objective metrics such as operative time, radiographic alignment, surgical accuracy, complication rates, or patient‐reported outcomes. Included studies also had to report the specific robotic systems used. A complete list of inclusion and exclusion criteria is presented in Table 1.

**TABLE 1:** Eligibility criteria.

Inclusion criteria	Exclusion criteria
Original research studies published in English	Review articles, conference abstracts, editorials, or technical notes
Focused on robotic‐assisted total knee arthroplasty (TKA)	Focused exclusively on non‐robotic arthroplasty procedures
Investigated learning curves using metrics such as operative time, radiographic alignment, surgical accuracy, complication rates, or patient‐reported outcomes	Lacked objective metrics relevant to the learning curve
Included data on robotic systems used for knee arthroplasty	

### 2.3 Data Extraction

Data extraction was performed independently by 2 reviewers on distinct spreadsheets, capturing relevant variables from each included study. Extracted information included study characteristics, the robotic system used, sample sizes, and reported outcomes such as operative time, radiographic alignment, complications, and PROMs. Where necessary data were unavailable, corresponding authors were contacted to obtain additional information. A full list of extracted variables can be found in Table S2. Any discrepancies in data extraction were resolved through discussion and consensus between reviewers.

### 2.4 Data Synthesis

Given the heterogeneity of study designs, robotic platforms, outcome measures, and definitions of proficiency, a meta‐analysis was not feasible. Instead, a qualitative synthesis was conducted, supplemented with weighted means, by sample size, where appropriate. Given substantial variability in these definitions and lack of access to case‐level raw data, recalibration or standardization of thresholds across studies or platforms was not feasible. LC thresholds were extracted and summarized by robotic platform, following SWiM guidelines [28]. For studies reporting ranges or surgeon‐level thresholds, weighted means were calculated on Microsoft Excel (Microsoft, 2024). Results are grouped thematically by outcome domain: operative time, radiographic accuracy, complication rates, and patient‐reported outcomes (PROMs).

### 2.5 Critical Appraisal

Risk of bias was assessed independently by two reviewers using the Methodological Index for Non‐Randomized Studies (MINORS) tool for observational studies [24], which evaluates methodological quality across 12 domains including study aim, follow‐up, and data collection. For randomized studies, the Cochrane Risk of Bias 2 (ROB‐2) tool [25] was used, which assesses bias across five key domains such as randomization, deviations from intended interventions, and outcome reporting (Table 2).

**TABLE 2:** Study outcomes.

Country	Study type	Study (year)	Robotic platform	Total raTKAs performed	Mean age of population (years)	Surgeon grade	LC threshold (Cases)	Primary LC metric(s)	PROMS reported	Complication rate (n, %)	Complication type(s)
USA	Retrospective cohort study	Ali et al. [29]	MAKO	120	66.1 ± 8.6	Non fellowship trained surgeon	Within 40	Operative time	Not reported	5, 4.16%	Cellulitis (*n* = 1), Acute Kidney Injury y (*n* = 1), Readmission for congestive heart failure exacerbation (*n* = 1), manipulation under anesthesia for decreased knee ROM (*n* = 2)
USA	Prospective observational cohort study	Bell et al. [30]	NAVIO	60	Not reported	Fellowship trained senior surgeon	29	Operative time	Not reported	0	N/A
New Zealand	Prospective cohort study	Bolam et al. [31]	ROSA	53	70.3 ± 8.6	Fellowship trained high volume surgeons	3 different surgeons = 15,5,6	Operative time	Not reported	4, 7.53%	Superficial wound site infections (*n* = 2), Post operative deep vein thrombosis (*n* = 1), Post op death due to unrelated cardiovascular event (*n* = 1)
Australia	Retrospective cohort study	Bourgeault‐Gagnon et al. [32]	VELYS	225	68.3 (SD not reported)	Not reported	Within 30	PE Insert Thickness	Not reported	Not reported	Not reported
USA	Retrospective cohort study	Chen et al. [33]	MAKO	5404	Not reported	Multicentre	Between 15 and 20	Operative time	Not reported	Not reported	Not reported
Australia	Retrospective observational cohort study	Collins et al. [34]	NAVIO	72	67 ± 10	Senior surgeon	No significant difference	Operative time and coronal alignment	Not reported	4, 5.56%	Manipulation under anesthesia (*n* = 2), Intraoperative tibial fracture (*n* = 1), Non‐fatal pulmonary embolism (*n* = 1)
Romania	Retrospective cohort study	Dragosloveanu et al. [24]	ROSA	39	69.15 ± 5.24	Experienced high‐volume orthopedic surgeons	3 surgeons = 6,3,4	Operative time	Not reported	0	N/A
China	Retrospective cohort study	Duan et al. [35]	JianJia	90	Learning group (*n* = 20) age: 70.50 ± 5.54, Proficiency group (*n* = 70) age: 67.80 ± 7.76	Clinician experienced in conventional surgical techniques using the same type of knee prosthesis	20	Operative time, surgical accuracy, complication rate	Not reported	0	N/A
Brazil	Retrospective cohort study	Ejnisman et al. [36]	ROSA & MAKO	321	70.74 ± 8.46	Initial stage and proficiency stage surgeons (proficiency meaning performed more than 10 RATKAs previously)	Approximately 30–40	Operative time	Not reported	Not reported	Not reported
USA	Retrospective Case Series	Grau et al. [37]	MAKO	132	Not reported	Senior surgeon	Within 40	Operative time	Not reported	Not reported	Not reported
Austria	Prospective observational cohort study	Haslhofer et al. [38]	MAKO	199	68.9 ± 11.5	Mixed (7 surgeons)	Estimated at 20	Operative time	Not reported	7, 3.9%	Not reported
South Korea	Prospective observational comparative cohort study	Jung et al. [20]	MAKO	50	70.3 ± 6.2	High‐volume surgeon (> 200 cases/year)	18	Operative time	Not reported	Not reported	Not reported
USA	Retrospective observational cohort study	Kang et al. [39]	ROSA & MAKO	95 ROSA, 115 MAKO	67 (IQR 60.5–73.5)	Fellowship‐trained surgeon	MAKO: 6, ROSA: 9	Operative time	No significance difference	ROSA: 3, 3.15%, MAKO: 11, 9.56%	ROSA: Arthrofibrosis requiring manipulation (*n* = 1), Periprosthetic fracture (*n* = 1), Revision (*n* = 1), MAKO: Deep vein thrombosis (*n* = 1), Arthrofibrosis requiring manipulation (*n* = 4), Periprosthetic fracture (*n* = 1), Wound dehiscence (*n* = 1), Revision (*n* = 4)
UK	Prospective single‐surgeon cohort study	Kayani et al. [40]	MAKO	60	64.8 ± 7.8	Senior surgeon	6	Operative time	Not reported	Not reported	Not reported
UK	Prospective cohort study	Kayani et al. [41]	MAKO	60	68.2 ± 7.0	High‐volume surgeon	7	Operative time	Not reported	Not reported	Not reported
Greece	Retrospective comparative cohort study	Kenanidis et al. [42]	ROSA	100	73.6 ± 6.3	High‐volume arthroplasty surgeon	73	Operative time	Not reported	0	N/A
India	Prospective observational cohort study	Londhe et al. [43]	Curvis Joint	40	67.3 ± 15.4	Experienced in conventional TKA	18	Operative time	Not reported	1, 2.5%	Wound dehiscence (*n* = 1)
India	Prospective observational comparative cohort study	Londhe et al. [44]	Curvis Joint	30	65.2 ± 12.8	Experienced surgical team	10	Operative time	Not reported	0	N/A
USA	Prospective multi‐center observational cohort study	Mahure et al. [45]	Tsolution One	115	65.9 ± 8.3	Fellowship‐trained orthopedic surgeons	Between 10 and 20	Operative time	Not reported	0	N/A
Israel	Retrospective cohort study	Masarwa et al. [46]	NAVIO	150	67.7 ± 8.9	Senior orthopedic surgeon	20	Operative time	RA‐TKA had significantly lower pain scores on post‐op day 1 (*p < 0.05*)	17, 11.3%	Post surgical infections (*n* = 3), Revision (*n* = 14)
USA	Retrospective observational comparative cohort study	Meghpara et al. [47]	MAKO	74	68.5 ± 8.2	Experienced arthroplasty surgeons	6	Operative time	Not reported	Not reported	Not reported
USA	Retrospective observational comparative cohort study	Morrisey et al. [48]	VELYS	66	68.2 ± 6.9	Senior arthroplasty surgeon	2	Operative time	No significant difference	No significant difference	Not reported
USA	Retrospective observational comparative cohort study	Naziri et al. [49]	MAKO	40	69.5 ± 8.3	Experienced arthroplasty surgeon	Within 20	Operative time	raTKA had improved ROM at 90 days (*p < 0.05*)	0	N/A
Spain	Prospective observational comparative cohort study	Neira et al. [50]	ROSA	90	72.4 ± 7.7	Three surgeons (1 senior, 2 junior)	43 (experienced), 61 (all surgeons)	Operative time	No significant difference	No significant differences	Not reported
USA	Retrospective observational cohort study	Patel et al. [51]	MAKO	682	64.2 ± 8.8	Fellowship‐trained arthroplasty surgeon	50 (initial improvement), 151–200 (max efficiency)	Operative time	Not reported	Not reported	Not reported
Germany	Prospective observational comparative cohort study	Probst et al. [52]	MAKO	351	70 ± 10.2	11 surgeons (6 experienced, 5 residents)	Between 18 and 55 (experience‐dependent)	Operative time, alignment accuracy	92% overall satisfaction at 90 days	2, 0.56%	Revision (*n* = 2)
Germany	Retrospective observational case–control study	Savov et al. [23]	NAVIO	70	64.4 ± 8.5	Senior surgeon	11	Operative time	Not reported	No increase in revision risk	Not reported
Austria	Prospective observational cohort study	Schopper et al. [53]	MAKO	31	70 ± 8.7	3 surgeons (1 experienced, 2 new)	9	Operative time	Not reported	No increase in revision risk	Not reported
France	Prospective cohort study	Shatrov et al. [54]	MAKO	50	67.6 ± 9.5	Experienced surgeon	30	Operative time	Not reported	Not reported	Not reported
Turkey	Retrospective observational study	Suzer et al. [55]	Not reported	52	median = 67; range = 55–84	Senior surgeons	11	Operative time	Not reported	4	Not stratified between raTKA and manual TKA
New Zealand	Retrospective observational cohort study	Tay et al. [56]	MAKO	101	68.2 ± 9.6	Senior arthroplasty consultants	16	Operative time	Not reported	5, 4.95%	Revision (*n* = 1), Manipulation under anesthesia (*n* = 1), Washouts (*n* = 3)
New Zealand	Retrospective cohort study	Tay et al. [57]	MAKO	152	64.9 ± 9.4	Senior surgeons	11	Operative time, planned vs. actual PE insert size difference	Not reported	0	N/A
Thailand	Randomized controlled trial	Thiengwittayaporn et al. [58]	NAVIO	75	69.0 ± 8.3	Experienced surgeon	7	Operative time	Not reported	Not reported	Not reported
Thailand	Retrospective observational study	Thongpulsawad et al. [59]	MAKO	110	Learning phase: 71.2 ± 6.8, Proficiency phase: 71.8 ± 7.5	Experienced surgeons	3 surgeons: 14,14,6	Operative time	Not reported	Not reported	Not reported
India	Retrospective observational comparative cohort study	Vaidya et al. [60]	NAVIO	75	Not reported	Senior arthroplasty surgeon +3 junior arthroplasty surgeons	Approximately 25	Operative time	Not reported	Not reported	Not reported
Belgium	Retrospective cohort study	Vanlommel et al. [25]	ROSA	90	68.7 ± 8.1	Orthopedic surgeons	3 surgeons: 10,6,11 cases	Operative time	Not reported	Minimal'	Not reported
Belgium	Retrospective observational cohort study	Vermue et al. [61]	MAKO	386	70.4 ± 8.6	Fellowship‐trained surgeons	Between 11 and 43	Operative time	Not reported	1, 0.26%	Persistent pain at tibial pin location (*n* = 1)
Belgium	Prospective observational cohort study	Vermue et al. [62]	OMNIBiotics	60	63.4 ± 11.8	Arthroplasty surgeon	Between 1 and 16	Operative time	Not reported	Not reported	Not reported
India	Retrospective observational cohort study	Weaver et al. [63]	CORI	500	67 ± 8.16	Not reported	6	Operative time	Not reported	11	Unintended bony over resection (*n* = 3), Soft tissue injury (*n* = 2), Robotic system hardware malfunction (*n* = 2), Software malfunction (*n* = 2), Superficial pin site infection (*n* = 1), periprosthetic fracture near the pin sites (*n* = 1)
China	Retrospective observational comparative cohort study	Zhang et al. [64]	HURWA	90	Surgeon 1: 68.30 ± 8.31, Surgeon 2: 67.87 ± 8.13, Surgeon 3: 64.87 ± 7.54	Experienced arthroplasty surgeons	Between 8 and 20	Operative time, bone cutting accuracy, and limb alignment	Not reported	Not reported	Not reported

## 3 Results

### 3.1 Study Selection

The initial database search identified 1801 studies. Following the removal of 482 duplicates, 1319 titles and abstracts were screened. Of these, 161 full‐text articles were assessed for eligibility, with 40 studies meeting the inclusion criteria and included in the final analysis. The study selection process is summarized in the PRISMA diagram (Figure 1).

### 3.2 Study Characteristics

The 40 included studies collectively evaluated 10,533 raTKA procedures published between 2019 and 2024. Ten robotic platforms were identified, with MAKO (Stryker) (19/40), NAVIO (Smith & Nephew) (6/40), and ROSA (7/40) (Zimmer Biomet) being the most frequently studied. Other systems included VELYS (2/40), Curvis Joint (2/40), Hurwa (1/40), OMNIbiotics (1/40), Cori (1/40), Tsolution One (1/40), and Jianjia (1/40). Surgeon experience varied considerably across studies, ranging from high‐volume arthroplasty specialists to general orthopedic surgeons with limited prior exposure to robotic systems. Full study characteristics can be found in Tables S3 and S2.

Of the included studies, 23 (57.5%) were retrospective and 17 (42.5%) were prospective in design. Of the 39 non‐randomized studies, 7 (18%) were judged to have a low risk of bias, 30 (77%) demonstrated moderate risk, and 2 (5%) were classified as high risk (Table S4). The remaining study, a randomized controlled trial, was deemed to have a low risk of bias (Table S5).

### 3.3 Operative Time

Operative time was the most frequently reported metric, described in 38 of the 40 studies, and was used as a primary marker of procedural familiarity. Most studies observed longer operative times in the initial cases, followed by a gradual reduction as experience increased. LC plateaus ranged from 2 to 73 cases, with a weighted mean reduction of 18.7 min between early and later cases. The absolute reduction in operative time varied from 2.4 to 33.3 min.

### 3.4 Surgical Accuracy and Radiographic Alignment

Surgical accuracy and component alignment were evaluated in 24 of the 40 included studies (60%), typically using postoperative radiographs to assess femoral and tibial component positioning in the coronal and sagittal planes. Of these, 21 studies (52.5%) reported no significant differences in alignment accuracy between the learning and proficiency phases or compared to mTKA.

Two studies [20, 41] reported a lower rate of alignment outliers in raTKA compared to mTKA. Outliers were typically defined as deviations greater than ±3° from the planned mechanical axis. Three studies [20, 37, 41] demonstrated improved implant positioning and overall limb alignment in the robotic‐assisted group. No studies reported a decline in accuracy during the learning phase.

### 3.5 Complication Rates

Complication data were available in 25 studies. Of these, 19 did not include a direct comparator group. Among the six comparative studies, four reported no significant difference in complication rates between raTKA and mTKA. One study [35] reported no complications in the raTKA group versus 14 events in the manual cohort, while another observed a slightly higher revision rate in the robotic group (14 vs. 11 cases) [46].

Reported complications varied in the raTKA cohorts, including arthrofibrosis (*n* = 5) [39], reduced postoperative range of motion requiring manipulation under anesthesia (*n* = 4) [29, 34], and wound‐related issues such as superficial infections or dehiscence (*n* = 5) [31, 41, 42, 43]. Robotic‐specific complications were also noted: tracker screw breakage (*n* = 2), robotic system failure (*n* = 4) [52], hardware or software malfunction (*n* = 4) [63], and pin‐site issues including infection or periprosthetic fracture (*n* = 3) [61, 63]. No studies reported a consistent increase in complication rates during the learning phase.

### 3.6 Patient‐Reported Outcomes

Patient‐reported outcome measures (PROMs) were evaluated in six studies. Four reported no significant differences between raTKA and manual procedures. However, two studies identified early functional benefits in the robotic cohort. Naziri et al. [49], in a single surgeon comparative analysis of the first 40 raTKA cases, found significantly improved range of motion at 90 days (*p* < 0.05), while Masarwa et al. [46], in a matched retrospective cohort study comparing manual and raTKAs, observed lower day‐one postoperative pain scores following raTKA (*p* < 0.05). Although limited in number, these findings suggest that robotic‐assisted techniques may offer short‐term improvements in recovery and comfort.

### 3.7 Platform Specific Analysis of Robotic Systems

The following section presents a system‐specific analysis of LC characteristics across the MAKO, NAVIO, and ROSA, which differ in their use of preoperative imaging, level of automation, and interoperative guidance. MAKO uses a CT‐based 3D model and robotic arm to guide bone cuts with haptic feedback. NAVIO generates a virtual model intraoperatively and assists resections using a handheld robotic burr. ROSA builds a 3D model from X‐rays and robotically positions cutting guides for surgeon‐led bone cuts [65].

### 3.8 MAKO System (Stryker)

Across 19 studies (*n* = 1913), the MAKO system had a weighted mean operative time of 82.0 min. On average, 34.2 procedures were needed to reach the learning curve threshold, representing the highest proficiency requirement among all platforms. Operative duration consistently decreased with increasing surgeon experience. For example, Ali et al. observed a reduction from 67.0 min in the first 20 cases to 59.3 min in subsequent ones (*p* < 0.0001), demonstrating a clear learning effect. Similar trends were reported by Chen et al. [35], although exact timings were not provided. Radiographic results were favorable across studies, with implant placement generally within ±2° of the mechanical axis and fewer alignment outliers compared to manual approaches [31, 35]. No deterioration in accuracy was observed during the learning phase. Haslhofer et al. [41] reported a 90‐day complication rate of 3.9%. None of the included MAKO studies reported data on patient satisfaction.

### 3.9 NAVIO System (Smith & Nephew)

Six studies (*n* = 502) evaluated the NAVIO platform. The weighted mean operative time was 81.9 min, and the average number of cases to reach proficiency was 18.4, the shortest among platforms. Bell et al. [30] documented a decline in operative time from 41.8 min in early cases to 31.1 min in later procedures. Collins et al. [34] reported an overall mean operative time of 41.2 min (SD 9.4). Although radiographic outcomes were reported less frequently, available data suggested improved mechanical alignment with fewer outliers when compared to manual surgery. Over 93% of NAVIO procedures achieved accurate coronal alignment, independent of case order [34]. Complication rates were low. Bell et al. [30] reported no adverse events, while Collins et al. [34] documented four complications across 72 cases. PROMs were not evaluated in any NAVIO studies.

### 3.10 ROSA System (Zimmer Biomet)

Eight studies (*n* = 577) assessed ROSA. The weighted mean operative time was 85.5 min, and the average number of cases required to reach proficiency was 29.5, placing it between MAKO and NAVIO in terms of training burden. Operative times decreased with greater familiarity. In a prospective, multi‐surgeon study, Bolam et al. [31] reported mean times falling from 114 min (SD 17) to 99 min (SD 18) as experience grew. Radiographic accuracy remained consistent, with no meaningful differences between planned and achieved implant positions. Bolam et al. [31] also reported five complications. No ROSA studies included PROM data.

### 3.11 MAKO and ROSA Combined

One study [37] included a pooled analysis of MAKO and ROSA cases (*n* = 321), reporting the longest weighted mean operative time of 161.3 min. The number of cases needed to achieve the learning curve was four, based on the authors' definition. However, outcomes were not stratified by individual platform, and as such, these findings should be interpreted with caution.

## 4 Discussion

### 4.1 Key Findings

This systematic review synthesizes evidence from 40 studies evaluating the LC in raTKA, encompassing 10,533 procedures across nine distinct robotic platforms. The number of cases required to achieve procedural proficiency varied considerably, ranging from 2 to 73 cases, with a weighted mean of 21.5 cases. Platform‐specific differences were prominent. NAVIO demonstrated the shortest LC with a mean of 18.4 cases, followed by ROSA at 29.5 cases. In contrast, MAKO exhibited the longest training phase with a weighted mean of 34.2 cases. These findings suggest that system design and workflow complexity play a key role in shaping the trajectory of skill acquisition.

### 4.2 Comparison With Existing Literature

Previous reviews have examined the LC in raTKA, but their generalized approach reduces applicability across different robotic platforms [66, 67, 68]. Zhang et al. [66] identified LC stabilization for operative time after 7–11 cases without evidence for a LC effect for component positioning accuracy nor difference in complication rates between early and late phases whilst Mancino et al. [67] provided a narrative review describing the different robotic platform characteristics available for raTKA. However, both treated robotic platforms as homogenous when citing LCs, overlooking system specific differences in workflow and technical complexity. Our review builds on these findings by separating results across individual systems, allowing for a deeper understanding of learning trajectories. Similarly, Vermue et al. [68] who estimated a LC of 6–20 cases, emphasized the lack of standardized metrics for defining proficiency such as the inconsistent definition of “operative *time*” which reduces comparability across the literature and compromises the generalisability of LC conclusions. Our review addresses these concerns by identifying where definitions and reporting varied, and by synthesizing data in a way that adjusts for platform level complexity.

### 4.3 Platform Architecture and Learning Curve Variation

The variability in LC thresholds reflects more than surgical volume and underscores the influence of platform architecture [26]. Murphy et al. [69] compared an image‐based system (MAKO) and an imageless system (OMNIBiotics) and found that despite requiring more tibial recuts, the OMNIBiotics system had a mean tourniquet operative time approximately 5 min shorter hinting at the more difficult ergonomics of the bulkier MAKO system may contribute to the increased time. In our review, operative times were nearly identical between the image based (MAKO) and imageless (NAVIO) systems (82 v 81.9 min). This indicates that comparable operative efficiency does not equate to comparable learning requirements and that image‐based systems may impose greater procedural and cognitive demands.

Imageless platforms such as NAVIO rely on intraoperative handheld mapping and avoid preoperative CT imaging, reducing procedural steps and preserving tactile feedback [29, 65], which may explain their shorter LCs. In contrast, MAKO requires preoperative imaging, segmentation, registration, and haptic boundary calibration, alongside a semi‐autonomous robotic arm that limits direct manual input [29]. These features likely contribute to its longer LC, particularly for surgeons less experienced with advanced image‐guided workflows [26]. ROSA, which combines image‐based planning with optical tracking and passive mechanical guidance, demonstrated an intermediate LC, reflecting its balance between technological assistance and procedural familiarity [29]. Overall, differences in the degree of automation, tactile engagement, and workflow integration inherent to each platform meaningfully shape the trajectory of skill acquisition in raTKA.

### 4.4 Clinical and Training Implications

The findings of this review highlight the need for competency‐based system specific training pathways in raTKA. The variation in LC durations across platforms suggests that platform architecture meaningfully influences learning time. For instance, MAKO requires preoperative CT imaging, virtual planning and active haptic boundaries which could contribute to its longer learning duration compared to the NAVIO (imageless) or ROSA (semi‐image based). Given these discrepancies, it may be beneficial to expose trainees to all major robotic platforms to ensure safe deployment across diverse surgical environments. Simulation based modalities can help deliver this training; virtual reality (VR) enables repetition of platform specific workflow and interface navigation which have been shown to improve performance. Goh et al. [70], in a focused review of surgical training modalities for knee arthroplasty, highlighted the utility of immersive VR platforms in enhancing procedural familiarity, cognitive mapping, and technical performance. This should be integrated with staged supervision and supported by structured objective feedback mechanisms. Echoing Soomro et al. [71], training frameworks should adopt multidimensional assessments incorporating intraoperative metrics, PROMs, and procedural flow. Hung et al. [72] further demonstrated, in the context of robot assisted radical prostatectomy, that machine learning algorithms applied to motion tracking data can classify surgical skills in real time based on tool and movement efficiency and path smoothness. Although applied in urology, this method is directly applicable to robotic TKA and represents a promising avenue for real time personalized trainee progression.

### 4.5 Determinants of Learning Curve Duration

The wide range in reported learning curve thresholds from 2 to 73 cases likely reflects heterogeneity within both the definitions of proficiency and the experience level of surgeons, rather than the intrinsic difficulty of robotic TKA. Morrisey et al. [48] reported the shortest threshold of 2 cases using VELYS in a single experienced surgeon, defining proficiency as tourniquet time plateau after initial cases, which represents a minimal definition of competence. In contrast, Kenanidis et al. [42] reported 73 cases for ROSA using time neutrality, defined as matching operative time to the same surgeon's manual TKA baseline, as the proficiency benchmark. This represents a more rigorous definition, requiring not just stabilization of operative time but equivalence to an established manual standard. Between these extremes, Dragosloveanu et al. [24] identified 3 to 6 cases for ROSA among three high volume surgeons, each performing over 300 TKA per year, in a dedicated orthopedic center with comprehensive preoperative training. In contrast, Neira et al. [50] found 61 cases for ROSA across three surgeons with mixed experience levels, including one with over 15 years of experience and two with less than 5 years, who received limited cadaveric training due to COVID 19 disruptions. These examples show that the apparent outliers stem from methodological choices, specifically the rigor of the definition of proficiency, whether surgeons were using simple time plateau or time neutrality benchmarks, their baseline experience and access to training support, and institutional context, rather than platform specific learning demands.

### 4.6 Emerging Role of Machine Learning in Skill Assessment

Machine learning (ML) presents a promising avenue for the objective assessment and enhancement of surgical proficiency in robotic‐assisted total knee arthroplasty (raTKA). We hypothesize that ML algorithms could be developed using comprehensive datasets including intraoperative metrics such as instrument motion trajectories, tool path efficiency, operative duration, and adherence to procedural workflow milestones. Analyzing trends within these data could help real‐time skill classification, predict progression along learning curves, and identify specific technical deficiencies. Although no current studies have validated ML‐based frameworks specifically in raTKA, analogous applications in other robotic‐assisted surgeries suggest their potential utility. Standardized capture of surgical performance metrics across robotic platforms will be imperative to enable the development of robust and generalisable ML models for the training and credentialing of surgeons in raTKA.

### 4.7 Strength and Limitations

While our study offers novel insights into the LCs surrounding raTKA, this review is limited by considerable heterogeneity in how LCs and proficiency were defined, modeled, and analyzed with wide variation in operative time cutoffs, analytical methods, and proficiency thresholds that prevented meaningful standardization and contributed to the broad range of reported LC durations.

Further, although operative time was the most frequently reported metric, its validity as a standalone measure of proficiency is limited. While operative time consistently decreased across studies, few investigations stratified complications, alignment accuracy, or other quality‐related outcomes by case order, making it unclear whether shorter procedures reflected genuine technical improvement. Most studies also relied on aggregate comparisons between robotic and manual TKA rather than evaluating how performance evolved across the LC. Additionally, operative time is heavily influenced by factors such as patient complexity, team composition, and institutional protocols, variables that were inconsistently reported, further limiting its utility in isolating surgeon learning. These inconsistencies reduce the reliability of pooled estimates and limit the generalisability of comparisons between platforms. Moreover, the absence of inferential analysis further hampers the interpretability of reported thresholds and likely contributes to the observed variability in LC durations. Moreover, the conclusions regarding stability in radiographic accuracy were similarly constrained by the inconsistent use and reporting of statistical methods across studies. While some employed *t*‐tests, ANOVA, or ICCs, others described outcomes qualitatively or did not specify their analytic approach. This lack of methodological uniformity restricts the strength of conclusions that can be drawn regarding radiographic outcomes across learning phases.

Surgeon and center‐level heterogeneity further complicate interpretation. Many studies omitted key details about operator experience, including prior robotic exposure, case volume, and training background. This made it impossible to subgroup by surgeon experience, despite its likely influence on learning efficiency. Participant heterogeneity was also evident in case selection, procedural complexity, and institutional resources. This variability reduces the ability to draw generalisable conclusions across platforms or surgical settings. This review also includes an imbalance in data representation among robotic systems, with some platforms having substantially more supporting studies. This variation may introduce bias, and while weighted means were used to partially mitigate this, potential bias remains. Publication and selection bias may also skew the findings. Most included studies were conducted in high‐volume, well‐resourced centers that may not reflect typical practice in lower‐volume units, and studies with null or negative results may be underrepresented. Consequently, the reported LC may present an idealized account of robotic implementation rather than the full range of real‐world experience. We also did not assess whether LCs varied over time, as methodological variability precluded formal analysis, underscoring the need for future studies to evaluate the impact of technological maturity and accumulated experience on learning efficiency.

### 4.8 Future Directions

Our findings reinforce the need for standardized endpoints and robust methodologies to accurately assess surgical LCs. Future studies should adopt structured approaches and define procedural proficiency using multidimensional metrics beyond operative time, including radiographic alignment, intraoperative complications, and recovery outcomes, all stratified by procedural sequence. This granularity could aim to distinguish genuine technical improvement from increasing familiarity. Standardization of procedural definitions, performance metrics, and outcome measures is essential to facilitate competency‐based training and objective assessment of surgical learning curves. Defining uniform benchmarks across studies and institutions allows for consistent identification of when proficiency is achieved, thereby supporting the development of reproducible, data‐driven training pathways. Such standardization enables meaningful comparisons between robotic platforms, reduces subjective bias in assessing performance, and improves the interpretability of reported learning thresholds. Ultimately, it ensures that observed improvements in operative performance reflect genuine skill acquisition rather than institutional variability or inconsistent reporting practices.

## 5 Conclusion

This systematic review demonstrates that LCs in robotic‐assisted total knee arthroplasty are not uniform but highly dependent on the platform utilized. The number of cases required to reach procedural proficiency varies markedly, with systems such as NAVIO associated with shorter learning phases and MAKO requiring substantially more operative experience. While operative time consistently improves with familiarity, radiographic accuracy and complication rates remain stable throughout the learning period, suggesting that early adoption does not compromise surgical precision or safety. However, heterogeneity in study design, outcome definitions, and reporting standards limits direct comparisons across platforms. These findings emphasize the urgent need for standardized, multidimensional frameworks, incorporating quantitative and system‐specific LCs, to define surgical proficiency, informing evidence‐based credentialing and robotic training pathways. As robotic technology becomes increasingly embedded in orthopedic practice, ensuring structured, platform‐specific education will be essential to optimize both patient outcomes and surgical efficiency.

## Author Contributions

**Ryhan Divyang Patel:** searching, methodology, writeup – original draft, writeup – revisions. **Praneshraja Ganesaraja:** searching, methodology, writeup – original draft, writeup – revisions. **Kapil Sugand:** writeup – original draft. **Sree Kanakala:** writeup – original draft. **Indi Gupte:** writeup – original draft. **Srikar Reddy Namireddy:** conceptualisation, writeup – revisions, supervision. **Saran Singh Gill:** conceptualisation, writeup – revisions, supervision. All authors have read and approved the final submitted manuscript.

## Funding

The authors have nothing to report.

## Conflicts of Interest

The authors declare no conflicts of interest.

## Supporting information


**Table S1:** Search strings.
**Table S2:**. Summary of data extraction variables and corresponding definitions used in the systematic review. Variables include study identifiers, design characteristics, robotic platform details, learning curve metrics, and reported outcomes relevant to learning curve assessment.
**Table S2:** Extraction variables.
**Table S3:**. Study characteristics.
**Table S4:**. Summary of the results of the methodological quality assessment of non‐randomized studies using the Methodological Index for Non‐Randomized Studies (MINORS) criteria. This tool includes 12 items, each scored from 0 to 2, where 0 indicates the item was not reported, 1 indicates it was reported but inadequate, and 2 indicates it was reported and adequate. The items assessed are: (1) a clearly stated aim; (2) inclusion of consecutive patients; (3) prospective collection of data; (4) endpoints appropriate to the aim of the study; (5) unbiased assessment of the study endpoint; (6) follow‐up period appropriate to the aim of the study; (7) loss to follow‐up less than 5%; (8) prospective calculation of the study size; (9) adequate control group; (10) contemporary groups; (11) baseline equivalence of groups; and (12) adequate statistical analyses. The maximum total score is 24, with studies categorized as having low, moderate, or high risk of methodological bias based on overall score and reporting quality.
**Table S3:** MINORS criteria.
**Table S5:** Summary of the results of the risk of bias assessment for randomized controlled trials using the Cochrane RoB‐2 tool. The evaluation covers five domains: (1) bias arising from the randomization process, (2) bias due to deviations from intended interventions, (3) bias due to missing outcome data, (4) bias in measurement of the outcome, and (5) bias in selection of the reported result. Each domain is rated as Low, Some concerns, or High. An overall judgment was then determined based on the domain‐level assessments.
**Table S4:**: RoB‐2.

## Data Availability

Research data are not shared.
