# Supplementary material for: Platform‐Specific Learning Curves in Robotic‐Assisted Total Knee Arthroplasty: A Systematic Review
Source: Orthop Surg. 2026 Apr 16;18(6):1116–32. doi: 10.1111/os.70304 (PMC13238661; doi:10.1111/os.70304)
Supplement: Supplementary file 1 — Table S1: Search strings. Table S2:. Summary of data extraction variables and corresponding definitions used in the systematic review. Variables include study identifiers, design characteristics, robotic platform details, learning curve metrics, and reported outcomes relevant to learning curve assessment. Table S2: Extraction variables. Table S3:. Study characteristics. Table S4:. Summary of the results of the methodological quality assessment of non‐randomized studies using the Methodological Index for Non‐Randomized Studies (MINORS) criteria. This tool includes 12 items, each scored from 0 to 2, where 0 indicates the item was not reported, 1 indicates it was reported but inadequate, and 2 indicates it was reported and adequate. The items assessed are: (1) a clearly stated aim; (2) inclusion of consecutive patients; (3) prospective collection of data; (4) endpoints appropriate to the aim of the study; (5) unbiased assessment of the study endpoint; (6) follow‐up period appropriate to the aim of the study; (7) loss to follow‐up less than 5%; (8) prospective calculation of the study size; (9) adequate control group; (10) contemporary groups; (11) baseline equivalence of groups; and (12) adequate statistical analyses. The maximum total score is 24, with studies categorized as having low, moderate, or high risk of methodological bias based on overall score and reporting quality. Table S3: MINORS criteria. Table S5: Summary of the results of the risk of bias assessment for randomized controlled trials using the Cochrane RoB‐2 tool. The evaluation covers five domains: (1) bias arising from the randomization process, (2) bias due to deviations from intended interventions, (3) bias due to missing outcome data, (4) bias in measurement of the outcome, and (5) bias in selection of the reported result. Each domain is rated as Low, Some concerns, or High. An overall judgment was then determined based on the domain‐level assessments. Table S4:: RoB‐2. [file OS-18-1116-s001.docx]

Supplementary Material

| Table S1: Search Strings | |
| --- | --- |
| Medline (239) | |
| 1 | (arthroplasties, knee replacement or arthroplasties, replacement, knee or arthroplasty, knee or arthroplasty, knee replacement or arthroplasty, partial knee or arthroplasty, replacement, knee or arthroplasty, replacement, partial knee or arthroplasty, total knee or arthroplasty, unicompartmental knee or arthroplasty, unicondylar knee or knee arthroplasty or knee arthroplasty, partial or knee arthroplasty, total or knee arthroplasty, unicompartmental or knee arthroplasty, unicondylar or knee replacement arthroplasties or knee replacement arthroplasty or knee replacement, partial or knee replacement, total or knee replacement, unicompartmental or knee replacement, unicondylar or partial knee arthroplasty or partial knee replacement or replacement arthroplasties, knee or replacement arthroplasty, knee or replacement, total knee or total knee arthroplasty or total knee replacement or unicompartmental knee arthroplasty or unicompartmental knee replacement or unicondylar knee arthroplasty or unicondylar knee replacement).mp. |
| 2 | (curve, learning or learning curve or learning curves).mp. |
| 3 | 1 AND 2 |
| Embase (359) | |
| 1 | (arthroplasty, knee or knee arthroplasties or knee reconstruction or reconstruction, knee or (arthroplasty, replacement, knee or knee joint replacement or knee joint replacements or knee replacement arthroplasty or knee replacements) or (knee arthroplasty, total or knee replacement, total or total knee joint replacement or total knee replacement or total knee replacement arthroplasty) or (TKA or UKA or (Unico* and Knee and (Replacement* or Arthroplast*)))).mp. [mp=title, abstract, heading word, drug trade name, original title, device manufacturer, drug manufacturer, device trade name, keyword heading word, floating subheading word, candidate term word] |
| 2 | (learn* and Curve*).mp. [mp=title, abstract, heading word, drug trade name, original title, device manufacturer, drug manufacturer, device trade name, keyword heading word, floating subheading word, candidate term word] |
| 3 | 1 AND 2 |
| Central (14) | |
| 1 | MeSH descriptor: [Arthroplasty, Replacement, Knee] explode all trees |
| 2 | MeSH descriptor: [Learning Curve] explode all trees |
| 3 | (Learn* AND Curve*) |
| 4 | 1 AND (2 OR 3) |
| Scopus (n=978) | |
| ( ( ( total OR partial ) AND knee AND ( replacement* OR arthroplast* ) ) OR tka OR tkr OR uka OR ukr ) AND ( learn* AND curve* ) AND ( LIMIT-TO ( SUBJAREA , "MEDI" ) ) AND NOT ( hip OR ai OR cnn OR "Neural Network" ) | |

**Table S2** Summary of data extraction variables and corresponding definitions used in the systematic review. Variables include study identifiers, design characteristics, robotic platform details, learning curve metrics, and reported outcomes relevant to learning curve assessment.

**Table S2: Extraction Variables**

| **Variable** | **Description** |
| --- | --- |
| First author and year of publication | Citation reference used to identify the study |
| Country of origin | Nation where the study was conducted |
| Study design | Observational or randomised; prospective or retrospective |
| Robotic system(s) used | Platform(s) evaluated (e.g., MAKO^™^, NAVIO^®^, ROSA^®^) |
| Sample size and number of cases | Total participants and procedures assessed |
| Learning curve metrics | Metrics used to define proficiency (e.g., operative time, inflection point) |
| Outcomes | Radiographic alignment, complication rates, PROMs |
| Key findings related to learning curve progression | Summary of conclusions regarding learning duration, trends, or plateau |

Table S3: Study Characteristics

| **Title** | **Author** | **Country** | **Study type** | **Conclusion** |
| --- | --- | --- | --- | --- |
| **Learning Curve of Robotic-Assisted Total Knee Arthroplasty for Non-Fellowship-Trained Orthopedic Surgeons** (29) | Ali et al. (2022) | USA | Retrospective cohort study | Robotic-assisted TKA can be safely performed by non-fellowship-trained surgeons after a short learning curve, with operative times approaching those of manual TKA within early cases. |
| **The successful implementation of the Navio robotic technology required 29 cases** (30) | Bell et al. (2022) | USA | Prospective observational cohort study | Robotic-arm-assisted TKA shows a short learning curve, with operative times decreasing after initial cases and the system becoming feasible for routine surgical use. |
| **Introduction of ROSA robotic-arm system for total knee arthroplasty is associated with a minimal learning curve for operative time** (31) | Bolam et al. (2022) | New Zealand | Prospective cohort study | The ROSA system shows a short learning curve for operative time and can be integrated into surgical teams without added patient risk. |
| **Robotic-Assisted Total Knee Arthroplasty Improves Accuracy and Reproducibility of the Polyethylene Insert Thickness Compared to Manual Instrumentation or Navigation: A Retrospective Cohort Study** (32) | Bourgeault-Gagnon et al. (2024) | Australia | Retrospective cohort study | ra-TKA offers improved reproducibility of surgical accuracy compared to other techniques, with a short learning curve to achieve consistent outcomes. |
| **Time-Based Learning Curve for Robotic-Assisted Total Knee Arthroplasty: A Multicenter Study** (33) | Chen et al. (2021) | USA | Retrospective cohort study | ra-TKA demonstrates a clear learning curve, with most surgeons achieving operative time consistency after early adoption. |
| **Initial Experience with the NAVIO Robotic-Assisted Total Knee Replacement-Coronal Alignment Accuracy and the Learning Curve** (34) | Collins et al. (2022) | Australia | Retrospective observational cohort study | The robotic-assisted system provides accurate coronal alignment in TKA and can be adopted without a significant learning curve. |
| **Initial Learning Curve for Robot-Assisted Total Knee Arthroplasty in a Dedicated Orthopedics Center** (35) | Dragosloveanu et al. (2023) | Romania | Retrospective cohort study | Experienced surgeons adapted rapidly to RA-TKA, with operative times decreasing and no increase in complications. |
| **Learning curve and short-term clinical outcomes of a new seven-axis robot-assisted total knee arthroplasty system: a propensity score-matched retrospective cohort study** (36) | Duan et al. (2023) | China | Retrospective cohort study | The seven-axis robotic system showed a short learning curve and was associated with improved alignment outcomes once proficiency was attained. |
| **Robot-assisted knee arthroplasty: Analyzing the learning curve and initial institutional experience.** (37) | Ejnisman et al. (2024) | Brazil | Retrospective cohort study | Surgical proficiency with robotic TKA appears dependent on individual surgeon adaptability, with operative times approaching those of manual TKA after initial adoption. |
| **Robotic arm assisted total knee arthroplasty workflow optimization, operative times and learning curve** (38) | Grau et al. (2019) | USA | Retrospective Case Series | With refined technique and workflow optimisation, robotic TKA can be performed efficiently, and surgical time need not be a barrier to adoption. |
| **No difference in surgical time and total theatre time between robotically assisted and computer assisted total knee arthroplasty** (39) | Haslhofer et al. (2024) | Austria | Prospective observational cohort study | Robotic TKA does not significantly increase surgical or total theatre time compared to computer-assisted TKA, and theatre efficiency can be maintained following adoption. |
| **Learning curve of robot-assisted total knee arthroplasty and its effects on implant position in asian patients: a prospective study** (40) | Jung et al. (2023) | South Korea | Prospective observational comparative cohort study | Robotic TKA offers more accurate and reproducible implant positioning than conventional manual TKA, with no significant difference in operative time once the learning curve is overcome. |
| **Comparison of learning curves and short-term outcomes: ROSA versus MAKO robotic-assisted total knee arthroplasty** (41) | Kang et al. (2024) | USA | Retrospective observational cohort study | Both ROSA and MAKO systems demonstrated short learning curves, with no differences in complications or functional outcomes between platforms. |
| **The learning curve associated with robotic-arm assisted unicompartmental knee arthroplasty: a prospective cohort study.** (42) | Kayani et al. (2018) | UK | Prospective single-surgeon cohort study | Robotic-arm assisted UKA was associated with a short learning curve for operative time and team confidence, without compromising implant positioning or increasing complication risk. |
| **Robotic-arm assisted total knee arthroplasty has a learning curve of seven cases for integration into the surgical workflow but no learning curve effect for accuracy of implant positioning** (43) | Kayani et al. (2019) | UK | Prospective cohort study | Robotic-arm assisted TKA was associated with a brief learning curve for operative time and team confidence, but showed no learning curve for implant positioning accuracy. It improved alignment outcomes without increasing complication rates compared to manual TKA. |
| **The learning curve to ROSA: cases needed to match the surgery time between a robotic-assisted and a manual primary total knee arthroplasty** (44) | Kenanidis et al. (2023) | Greece | Retrospective comparative cohort study | The study found that operative times for robotic and manual TKA equalised after a substantial case volume, indicating a prolonged learning curve for mastering the ROSA system. However, robotic adoption did not increase complication rates. |
| **Analysis of robot-specific operative time and surgical team anxiety level and its effect on alignment during robot-assisted TKA** (45) | Londhe et al. (2024) | India | Prospective observational cohort study | This study suggests that while operative times and surgical team anxiety improved with experience, there was no learning curve observed for implant positioning or limb alignment. Early concerns about increased surgical time with robotic assistance may diminish as proficiency is gained. |
| **Comparison of Time Taken in Conventional versus Active Robotic-Assisted Total Knee Arthroplasty** (46) | Londhe et al. (2024) | India | Prospective observational comparative cohort study | This study suggests that robotic-assisted total knee arthroplasty does not significantly increase operative time compared to manual techniques, potentially alleviating concerns about workflow delays during robotic integration. |
| **Learning curve for active robotic total knee arthroplasty** (47) | Mahure et al. (2022) | USA | Prospective multi-center observational cohort study | Active robotic total knee arthroplasty appears to be associated with a relatively short learning curve, primarily affecting surgical time. Importantly, this adjustment period did not impact patient-reported outcomes or complication rates. |
| **Short-term outcomes of an imageless robot-assisted total knee arthroplasty compared with a conventional method: A retrospective cohort study** (48) | Masarwa et al. (2022) | Israel | Retrospective cohort study | Imageless robot-assisted TKA offered modest short-term benefits in early recovery without increasing surgical duration, though longer-term advantages remain uncertain. |
| **The Ability of Robot-Assisted Total Knee Arthroplasty in Matching the Efficiency of Its Conventional Counterpart at an Orthopaedic Specialty Hospital** (49) | Meghpara et al. (2023) | USA | Retrospective observational comparative cohort study | Incorporating RA-TKA into a high-volume orthopaedic centre was associated with minimal increases in surgical timing and no delay in turnover, suggesting smooth integration without significant workflow disruption. |
| **Transition to Robotic Total Knee Arthroplasty With Kinematic Alignment is Associated With a Short Learning Curve and Similar Acute-Period Functional Recoveries.** (50) | Morrisey et al. (2023) | USA | Retrospective observational comparative cohort study | Transitioning to kinematically aligned robotic TKA resulted in comparable early recovery to traditional methods, with improved mid-term flexion and a short technical learning curve. Long-term benefits remain to be defined. |
| **Making the transition from traditional to robotic-arm assisted TKA: What to expect? A single-surgeon comparative-analysis of the first-40 consecutive cases** (51) | Naziri et al. (2019) | USA | Retrospective observational comparative cohort study | Robotic-arm assisted TKA demonstrated a rapid learning curve with shorter length of stay and improved short-term range of motion compared to traditional TKA, while maintaining comparable complication rates and functional outcomes. |
| **Analysis of the Initial Learning Curve for Robotic-Assisted Total Knee Arthroplasty Using the ROSA Knee System** (52) | Neira et al. (2024) | Spain | Prospective observational comparative cohort study | Surgeon experience with manual TKA significantly influences the learning curve for robotic-assisted TKA. While operative times for raTKA are initially longer, experienced surgeons can reach comparable efficiency to manual techniques after a defined learning phase. |
| **Robotic-assisted total knee arthroplasty: Is there a maximum level of efficiency for the operating surgeon?** (53) | Patel et al. (2022) | USA | Retrospective observational cohort study | This study highlights a progressive improvement in surgical efficiency with increased experience using robotic-assisted TKA, eventually reaching a plateau. These findings offer valuable insight for surgeons integrating robotic technology into practice. |
| **Learning curves of robotic technology in an orthopedic teaching hospital.** (54) | Probst et al. (2022) | Germany | Prospective observational comparative cohort study | Robotic-assisted knee arthroplasty demonstrated a learning curve in surgical duration, but not in precision, complication rates, or patient satisfaction. These findings support its use as an effective training platform in residency without compromising outcomes. |
| **Imageless robotic handpiece-assisted total knee arthroplasty: a learning curve analysis of surgical time and alignment accuracy** (55) | Savov et al. (2021) | Germany | Retrospective observational case–control study | Imageless robotic-assisted TKA demonstrated a short learning curve for surgical time, while accuracy in implant positioning remained consistent from the outset. These findings highlight the precision and adaptability of the robotic system, independent of user experience. |
| **The learning curve in robotic assisted knee arthroplasty is flattened by the presence of a surgeon experienced with robotic assisted surgery** (56) | Schopper et al. (2023) | Austria | Prospective observational cohort study | Support from an experienced surgeon shortens the learning curve for new teams adopting robotic-assisted knee arthroplasty, highlighting the benefit of early mentorship during implementation. |
| **How long does image based robotic total knee arthroplasty take during the learning phase? Analysis of the key steps from the first fifty cases** (57) | Shatrov et al. (2023) | France | Prospective cohort study | Significant improvements in total operating time occur early in the learning phase of MAKO robotic-assisted TKA, with key reductions seen after around 30 cases. The system is not time-intensive for the surgical team. |
| **Comparison of conventional and robotic knee arthroplasty results: A retrospective observational study.** (58) | Suzer et al. (2023) | Turkey | Retrospective observational study | The standard combined spinal epidural anaesthesia with controlled hypotensive anaesthesia provided effective anaesthetic conditions for most robotic TKA cases, despite a small proportion requiring conversion to general anaesthesia. Adjustments to extend anaesthesia duration may improve its suitability for robotic procedures. |
| **Robotic-arm assisted total knee arthroplasty has a learning curve of 16 cases and increased operative time of 12min** (59) | Tay et al. (2022) | New Zealand | Retrospective observational cohort study | The introduction of robotic-arm assisted TKA led to a learning curve marked by increased operative times and challenges in polyethylene insert sizing. However, patient outcomes were similar between learning and proficiency phases, providing useful expectations for surgeons adopting this technology. |
| **Robotic-arm assisted unicompartmental knee arthroplasty system has a learning curve of 11 cases and increased operating time** (60) | Tay et al. (2023) | New Zealand | Retrospective cohort study | The introduction of a robotic-arm assisted system for UKA showed a short learning curve affecting operative time and insert sizing, but no impact on implant survival at early follow-up. This suggests the system may be especially beneficial for surgeons with lower UKA volumes. |
| **Imageless robotic-assisted total knee arthroplasty accurately restores the radiological alignment with a short learning curve: a randomized controlled trial** (61) | Thiengwittayaporn et al. (2021) | Thailand | Randomised controlled trial | Imageless robotic-assisted TKA offers improved accuracy in knee alignment and implant positioning compared to manual TKA, with fewer alignment outliers. It is associated with a short learning curve, making it an appealing option for total knee arthroplasty. |
| **A novel robotic surgical assistant for total knee arthroplasty has a learning curve ranging from 6 to 14 cases and exhibits high accuracy in tibial bone cuts** (62) | Thongpulsawad et al. (2024) | Thailand | Retrospective observational study | The ROSA Knee System can be adopted after a modest learning curve, leading to improved operative efficiency. It provides accurate and reproducible bone cuts and alignment, supporting its effective integration into surgical workflows. |
| **Learning curve for robotic assisted total knee arthroplasty: our experience with imageless hand-held Navio system** (63) | Vaidya et al. (2023) | India | Retrospective observational comparative cohort study | Our experience shows that robotic-assisted total knee arthroplasty involves a learning curve, with operative times improving over consecutive cases until they become comparable to manual methods. Understanding this learning phase may help surgeons adopt robotic technology more confidently and achieve better outcomes. |
| **The initial learning curve for the ROSA Knee System can be achieved in 6-11 cases for operative time and has similar 90-day complication rates with improved implant alignment compared to manual instrumentation in total knee arthroplasty** (64) | Vanlommel et al. (2021) | Belgium | Retrospective cohort study | This robotic cutting guide system demonstrates a rapid learning curve for operative times and can be integrated efficiently into surgical practice. It offers comparable complication rates and improved component positioning compared to manual techniques. Continued use is expected to further enhance proficiency and outcomes. |
| **Robot-assisted total knee arthroplasty is associated with a learning curve for surgical time but not for component alignment, limb alignment and gap balancing** (65) | Vermue et al. (2022) | Belgium | Retrospective observational cohort study | Robot-assisted TKA involves a learning curve for operative time that varies depending on the surgeon’s experience, but no learning curve is observed for implant positioning, limb alignment, or joint balancing. These findings highlight that while efficiency improves with experience, precision is maintained throughout the learning phase. |
| **The learning curve of imageless robot-assisted total knee arthroplasty with standardised laxity testing requires the completion of nine cases, but does not reach time neutrality compared to conventional surgery.** (66) | Vermue et al. (2023) | Belgium | Prospective observational cohort study | The introduction of an imageless robotic system for total knee arthroplasty involves a learning curve based on operative time, with robotic procedures taking longer than manual surgery. However, there is no learning curve effect on coronal limb or implant alignment. This information aids surgeons in understanding the integration and workflow of this robotic system in clinical practice. |
| **Complications and Learning Curve Associated with an Imageless Burr-Based (CORI) Robotic-Assisted Total Knee Arthroplasty System: Results from First 500 Cases.** (67) | Weaver et al. (2024) | India | Retrospective observational cohort study | The study describes the learning curve and complication profile for a newly introduced imageless, burr-based robotic-assisted total knee arthroplasty system. This information aims to assist surgeons in adopting this technology by highlighting potential challenges and guiding safe integration into clinical practice. |
| **Learning curve analysis of robotic-assisted total knee arthroplasty with a Chinese surgical system** (68) | Zhang et al. (2023) | China | Retrospective observational comparative cohort study | The study identified a learning curve for robotic-assisted TKA using the HURWA system related to operative time, with no learning curve effect for bone cutting accuracy or limb alignment. More experienced surgeons adapted faster, but overall, the HURWA robot improved limb alignment compared to manual TKA regardless of surgeon experience. |

**Table S4** Summary of the results of the methodological quality assessment of non-randomised studies using the Methodological Index for Non-Randomized Studies (MINORS) criteria. This tool includes 12 items, each scored from 0 to 2, where 0 indicates the item was not reported, 1 indicates it was reported but inadequate, and 2 indicates it was reported and adequate. The items assessed are: (1) a clearly stated aim; (2) inclusion of consecutive patients; (3) prospective collection of data; (4) endpoints appropriate to the aim of the study; (5) unbiased assessment of the study endpoint; (6) follow-up period appropriate to the aim of the study; (7) loss to follow-up less than 5%; (8) prospective calculation of the study size; (9) adequate control group; (10) contemporary groups; (11) baseline equivalence of groups; and (12) adequate statistical analyses. The maximum total score is 24, with studies categorized as having low, moderate, or high risk of methodological bias based on overall score and reporting quality.

| **Table S3: MINORS Criteria** | | | | | | | | | | | | | | |
| --- | --- | --- | --- | --- | --- | --- | --- | --- | --- | --- | --- | --- | --- | --- |
| **Study (Year)** | **1** | **2** | **3** | **4** | **5** | **6** | **7** | **8** | **9** | **10** | **11** | **12** | **Total** | **Overall Judgment** |
| Ali et al. (2022) | 2 | 2 | 2 | 2 | 1 | 2 | 2 | 2 | 1 | 1 | 1 | 2 | 20 | Moderate |
| Bell et al. (2022) | 2 | 2 | 2 | 1 | 1 | 2 | 2 | 2 | 0 | 1 | 1 | 1 | 17 | High |
| Bolam et al. (2022) | 2 | 2 | 2 | 2 | 2 | 2 | 2 | 2 | 1 | 1 | 1 | 2 | 21 | Moderate |
| Bourgeault-Gagnon et al. (2024) | 2 | 2 | 2 | 2 | 2 | 2 | 2 | 2 | 2 | 2 | 1 | 2 | 23 | Low |
| Chen et al. (2023) | 2 | 2 | 2 | 2 | 2 | 2 | 2 | 2 | 2 | 2 | 2 | 2 | 24 | Low |
| Collins et al. (2022) | 2 | 2 | 2 | 2 | 2 | 2 | 2 | 2 | 1 | 1 | 1 | 2 | 21 | Moderate |
| Dragosloveanu et al. (2023) | 2 | 2 | 2 | 1 | 1 | 1 | 2 | 2 | 0 | 1 | 1 | 1 | 16 | High |
| Duan et al. (2023) | 2 | 2 | 2 | 2 | 2 | 2 | 2 | 2 | 2 | 2 | 1 | 2 | 23 | Low |
| Ejnisman et al. (2024) | 2 | 2 | 2 | 1 | 1 | 2 | 2 | 2 | 1 | 1 | 1 | 1 | 18 | Moderate |
| Grau et al. (2019) | 2 | 2 | 2 | 2 | 2 | 2 | 2 | 2 | 1 | 1 | 1 | 2 | 21 | Moderate |
| Haslhofer et al. (2024) | 2 | 2 | 2 | 2 | 1 | 1 | 2 | 0 | 2 | 2 | 2 | 2 | 20 | Moderate |
| Jung et al. (2023) | 2 | 2 | 1 | 2 | 1 | 2 | 2 | 0 | 2 | 2 | 2 | 2 | 20 | Moderate |
| Kang et al. (2024) | 2 | 2 | 2 | 2 | 1 | 1 | 2 | 0 | 1 | 2 | 1 | 2 | 18 | Moderate |
| Kayani et al. (2018) | 2 | 2 | 2 | 2 | 2 | 2 | 2 | 2 | 2 | 2 | 2 | 2 | 24 | Low |
| Kayani et al. (2019) | 2 | 2 | 2 | 2 | 2 | 2 | 2 | 2 | 2 | 2 | 2 | 2 | 24 | Low |
| Kenanidis et al. (2023) | 2 | 2 | 1 | 2 | 1 | 1 | 2 | 0 | 2 | 2 | 2 | 2 | 19 | Moderate |
| Londhe et al. (2024) | 2 | 2 | 2 | 2 | 1 | 1 | 2 | 0 | 2 | 2 | 2 | 2 | 20 | Moderate |
| Londhe et al. (2024) | 2 | 2 | 2 | 2 | 1 | 1 | 2 | 0 | 2 | 2 | 2 | 2 | 20 | Moderate |
| Mahure et al. (2022) | 2 | 2 | 2 | 2 | 1 | 1 | 2 | 0 | 1 | 2 | 1 | 2 | 18 | Moderate |
| Masarwa et al. (2022) | 2 | 2 | 1 | 2 | 1 | 1 | 2 | 0 | 2 | 2 | 2 | 2 | 19 | Moderate |
| Meghpara et al. (2023) | 2 | 2 | 2 | 2 | 1 | 1 | 2 | 1 | 2 | 2 | 2 | 2 | 21 | Moderate |
| Morrisey et al. (2023) | 2 | 2 | 1 | 2 | 1 | 1 | 2 | 0 | 2 | 2 | 2 | 2 | 19 | Moderate |
| Naziri et al. (2019) | 2 | 2 | 1 | 2 | 1 | 1 | 2 | 0 | 2 | 1 | 2 | 2 | 18 | Moderate |
| Neira et al. (2024) | 2 | 2 | 1 | 2 | 1 | 2 | 2 | 0 | 2 | 2 | 2 | 2 | 20 | Moderate |
| Patel et al. (2022) | 2 | 2 | 1 | 2 | 1 | 2 | 2 | 0 | 2 | 2 | 2 | 2 | 20 | Moderate |
| Probst et al. (2022) | 2 | 2 | 1 | 2 | 1 | 2 | 2 | 0 | 2 | 2 | 2 | 2 | 20 | Moderate |
| Savov et al. (2021) | 2 | 2 | 1 | 2 | 1 | 1 | 2 | 0 | 2 | 2 | 2 | 2 | 19 | Moderate |
| Schopper et al. (2023) | 2 | 2 | 2 | 2 | 2 | 2 | 2 | 2 | 2 | 2 | 2 | 2 | 24 | Low |
| Shatrov et al. (2023) | 2 | 2 | 2 | 2 | 1 | 1 | 2 | 1 | 2 | 2 | 2 | 2 | 21 | Moderate |
| Suzer et al. (2023) | 2 | 2 | 1 | 2 | 1 | 1 | 2 | 0 | 2 | 2 | 2 | 2 | 19 | Moderate |
| Tay et al. (2022) | 2 | 2 | 1 | 2 | 1 | 1 | 2 | 0 | 2 | 2 | 2 | 2 | 19 | Moderate |
| Tay et al. (2023) | 2 | 2 | 1 | 2 | 1 | 2 | 2 | 0 | 2 | 2 | 2 | 2 | 20 | Moderate |
| Thongpulsawad et al. (2024) | 2 | 2 | 1 | 2 | 1 | 1 | 2 | 0 | 2 | 2 | 2 | 2 | 19 | Moderate |
| Vaidya et al. (2023) | 2 | 2 | 1 | 2 | 1 | 2 | 2 | 0 | 2 | 2 | 2 | 2 | 20 | Moderate |
| Vanlommel et al. (2021) | 2 | 2 | 1 | 2 | 1 | 2 | 2 | 0 | 2 | 2 | 2 | 2 | 20 | Moderate |
| Vermue et al. (2022) | 2 | 2 | 1 | 2 | 1 | 1 | 2 | 0 | 2 | 2 | 2 | 2 | 19 | Moderate |
| Vermue et al. (2023) | 2 | 2 | 1 | 2 | 1 | 1 | 2 | 0 | 2 | 2 | 2 | 2 | 19 | Moderate |
| Weaver et al. (2024) | 2 | 2 | 1 | 2 | 1 | 2 | 2 | 0 | 2 | 2 | 2 | 2 | 20 | Moderate |
| Zhang et al. (2023) | 2 | 2 | 2 | 2 | 2 | 2 | 2 | 2 | 2 | 2 | 2 | 2 | 24 | Low |

**Table S5** Summary of the results of the risk of bias assessment for randomised controlled trials using the Cochrane RoB-2 tool. The evaluation covers five domains: (1) bias arising from the randomisation process, (2) bias due to deviations from intended interventions, (3) bias due to missing outcome data, (4) bias in measurement of the outcome, and (5) bias in selection of the reported result. Each domain is rated as Low, Some concerns, or High. An overall judgment was then determined based on the domain-level assessments.

| **Table S4: RoB-2** | | | | | | |
| --- | --- | --- | --- | --- | --- | --- |
| **Study** | **Bias from randomisation process** | **Bias due to deviations from intended interventions** | **Bias due to missing outcome data** | **Bias in measurement of the outcome** | **Bias in selection of the reported result** | **Overall Risk of Bias** |
| Thiengwittayaporn et al. (2021) | Low | Low | Low | Low | Low | Low |
